# Sustaining the future: How green capabilities and digitalization drive sustainability in modern business

**DOI:** 10.1016/j.heliyon.2024.e24158

**Published:** 2024-01-07

**Authors:** Zeying Li, Saad Rasool, Mustafa Fedai Cavus, Waseem Shahid

**Affiliations:** a Department of Computer Science and Engineering, Hanshan Normal University, Chaozhou, PR China; b School of Business, Shantou University, Shantou, PR China; c Department of Computer Science, Concordia University Chicago, 7400 Augusta St, River Forest, IL, 60305, United States; d Faculty of Economics and Administrative Sciences, Osmaniye Korkut Ata University, Osmaniye, Turkey; e Faculty of Business and Management, Universiti Teknologi MARA (UiTM), Selangor, Malaysia

**Keywords:** Digitization, Business sustainability, Competitive advantage, Environmental and financial performance, Technology adoption, Supply chain management

## Abstract

In recent years, the unprecedented growth in environmental vulnerabilities has made the firms realize the need for environmental protection. With this, the rapid surge for ecological preservation has made worldwide businesses divert their focus toward greener practices that ensure the firm’s financial and environmental performance. This study examines the relationships between green management strategies (green dynamic capabilities, internal green supply chain management and green technology adoption), and organizational outcomes, specifically environmental and financial performance. The data was collected from the 471 employees working in the manufacturing firms. Utilizing the Structural Equation Modeling (SEM) method via Smart-PLS, our findings show the importance of integrating green practices in supply chain management, dynamic capabilities, and technology adoption to enhance both environmental and financial outcomes under the moderating role of industry dynamism and green knowledge acquisition.

## 1 Introduction

Industrialization, a critical driver of the country’s economic progress today, requires global firms to work for environmental safety [1]. As industrial activities radially contribute to ecosystem destruction [2], worldwide ecological changes call for firms to embrace environmental measures, ensuring firms' sustainable performance. The growing climatic problems considerably damage natural habitats [3]. This alteration demands urgent considerations to ensure ecological protection [4].

With environmental issues becoming the global consensus [5] today, many businesses have adopted green developments to improve their performance. Climate change, an inevitable problem, requires firms to go green to leverage firms' performance [6]. As the firms are the basic unit for the country’s progress, their financial and environmental performance [7] ensures nature’s safety, which is the most critical aspect of firms’ performance [2].

The main motive of the firms is to strengthen the country’s economy by minimizing energy usage [8]. Recently, the excessive use of natural resources has made Chinese manufacturing enterprises face difficulty in coordinating the firms' performances (i.e., financial and environmental performance) [9]. Developing a cost-effective green dynamic capabilities (GDC) is key to achieving environmental performance (EVP) and financial performance (FP). Manifesting this issue, many businesses have adopted green capabilities as a pathway to improving the firms' FP and EVP [10]. The green dynamic capability enables the firms to compete on its environmental strengthens that nurture the firms' performance [11]. It allows firms to respond to changing market conditions by adopting eco-friendly resources and assets. The green dynamic capabilities includes employees' green skills, competencies, knowledge, abilities, principles, and behaviors [12]. It refers to the company’s ability to adapt to the changing market dynamics related to sustainability, customer demand, and regulations through green technology, skills, and competencies [13]. It explores and exploits the green opportunities that lead the firms to green innovation transition and competitiveness. Green enterprises developing eco-friendly capabilities yield green competitive adoption (GCA) [14]. It enables firms to discover environmentally friendly opportunities that drive firms' performance [15].

Green organizations respond to industry changes to gain a superior competitive advantage. Therefore, amidst high environmental uncertainties, today’s organizations have minimized the risk of market disruptions and climate change by pooling their inventories with green developments [16]. Today, the rapid evolution of technologies has significantly encouraged businesses to transform their technological, economic, and sociocultural phenomenon [17]. At the policy level, it is widely accepted that organizations may face complexities in integrating digitalization with green capabilities [18]. In the era of marked technological advancement, businesses may strive hard to adapt to the changing market conditions. They may encounter various challenges that need to be navigated effectively to gain superior firm performance and competitiveness. Digitalization requires firms to model their supply chain operations toward new change. It is a complex task as it is often met with resistance, lack of capability, inadequate green resources, privacy issues, freedom of choice, etc. [19]. These challenges of the intertwining digitalization and green dynamic capabilities make it hard for the firms to measure it empirically, as well as its underlying relationship with the firms' performance. Enabling the processing role of GDC, the employment of green technologies has also been argued to enhance EVP [20]. Green problems, central to economic globalization, require reducing the impact of increasing industrialization [21] with green technology. Khalil et al. [22] state that Internal Green Supply Chain Management (IGSCM) and Green Technology Adoption (GTA) are essential drivers of firms' performance. Accordingly, Sun et al. [23] show that despite China’s economic growth, China’s manufacturing is still experiencing a disadvantage of low performance. This deficiency demands creating an absolute green first-mover advantage that can help the companies improve the organization’s performance [24]. The current study provides a deeper understanding of the green factors needed to optimize the firm’s performance and competitiveness.

The firms constantly need to adapt to the changing market conditions [25]. In this regard, the GCA is a principal factor in dealing with the sensitivity of global market demands. Further, it is suggested it not only makes the firm adapt to the changing environment but also helps achieve EVP [16]. In manufacturing, paying attention to cultivating green competitiveness today demands that firms improve their learning that creates, shares, and transforms the firm’s eco-culture. Green Knowledge Acquisition (GKA), a learning motivation for management, supports the firm’s performance [7]. As knowledge is critical to creating value for firms, this study adopts the notion of green knowledge acquisition (GKA) to understand its impact on firms' performance. As the green competitive advantage (GCA) and green knowledge acquisition (GKA) have been prevalent in manufacturing enterprises for a long time [26], this study establishes these constructs of novelty to inspect the firms' performance.

The study aims to investigate the green factors (i.e., GDC, IGSCM, GTA) that are valuable to leveraging the firm’s financial and environmental performance under the mediated moderated model. It is worth noting that these connotations and measurements significantly cultivate the firms' performance and competitiveness. The factors considered in this study affect Chinese manufacturing businesses. The manufacturing industry is the reason behind the country’s development. In the last few years, the scale of the Chinese manufacturing industry has gained hyped, and its contribution to GDP has grown tremendously. However, the dilemma is still confusing regarding green adoption. Hence, to fill the research gap, it is essential to have a comprehensive view of the current market situation in China [27]. Therefore, this study adopts a profound conceptual framework of green capabilities, technologies, and high-end supply chain internal efficiencies, thus improving the firms' performance and competitiveness.

The shortcoming of the growing environmental problems has severely affected Chinese manufacturing firms, so for competitive development, this model is a significant one that may lead to massive transformations. Significantly, this study in Chinese manufacturing firms will be a momentous practical guide that will enrich future studies to gain GCA from the perspective of GDC, IGSCM, GTA, GKA, and ID. All these factors will promote the efficient allocation of resources while optimizing the firms' FP and EVP.

## 2 Theoretical background and hypothesis development

### 2.1 Green dynamic capabilities

In recent years, ecological uncertainty has raised monumental environmental problems for manufacturing businesses. The climate issues seemingly becoming more urgent today require the firms to respond to the increasing market changes. This deficiency suggests the concept of green dynamic capabilities to address the most pressing issue of ecological damages influencing the firms' FP [28]. The green dynamic capabilities refer to the company’s ability to sense ecological opportunities [29]. These dynamic capabilities include new environmental technologies and innovations. GDC enables the firms to adopt ecological skills and competencies that make the firms improve their performance. These capabilities in manufacturing allow businesses to transform their traditional practices into ecological activities [30]. Sarfraz et al. [27] argue that these proactive capabilities greatly influence the firm’s FP by bringing change in the firm’s culture. In the manufacturing economy, the firms' economic success heavily depends on their ability to generate high profits. The GDC maximizes the company’s profits while reducing the cost associated with environmental risk. As the firm’s objective is to gain economic growth, the GDC brings positive change in organizational ecological activities, thus supporting high FP [31].

The company effectively mobilizes firms' resources to tap the opportunities that leverage firms' ecological competitiveness. The GDC makes the firms offer eco-friendly products that set the business apart from their competitors [32]. Demonstrating the commitment to these capabilities enables manufacturing firms to adhere to the environmental standards that play a vital role in GCA [29]. According to the resource-based view, the rare and non-imitable organizations' capabilities provide a superior competitive advantage [33]. The capability concept in the management literature has been for years. Therefore, valuing the notion of RBV, in recent years, the green dynamic capabilities have strategically emerged as the most valued capability involving green design, production, recycling, and green supply chain [34]. In a highly turbulent manufacturing market, green transformations are adjusted to complement firms' competitive position [35]. It motivates firms to adopt green operations that predict consumer preferences, market demands, and requirements [36]. Indeed, sensing the organization’s capacity and utilizing the signals for change, the GDC has a strong propensity to tackle the challenges and opportunities necessary to gain a green advantage.

Promoting resource capability can bring a differentiating advantage for the firms. GDCs, hard for competitors to reach, improve the firms' EVP [37]. The environmental orientation demands firms' adopt the green dynamic capability to gain EVP [38]. Achieving environmental performance is a significant goal that has caused worldwide manufacturing to understand the importance of market changes. The manufacturing industry, experiencing intense pressure from the stakeholders today, emphasizes coping with the changing market environment through GDC. The dynamic capabilities stimulating the idea of greenness compel the company to improve EVP [39]. The GDCs prevented in the previous literature are essential to the firms' EVP [40]. Therefore, in manufacturing, the firms must adopt capabilities that enhance their EVP. Therefore, based on this, we conclude:

H1(a1) Green dynamic capabilities have positive and significant impact on financial performance.

H1(a2) Green dynamic capabilities have positive and significant impact on green competitive advantage.

H1(a3) Green dynamic capabilities have positive and significant impact on environmental performance.

### 2.2 Internal green supply chain management

To address the growing environmental concerns today, stakeholders emphasize the need to focus on the firm’s internal operations. Internal Green Supply Chain Management is a widely discussed topic in the manufacturing literature that refers to the firm’s decision to act ecologically [41]. As economic development in China is gradually becoming greener [42], the regional economic structure demands that organizations transform their activities to gain financial growth [43]. The IGSCM is a critical factor influencing the firms' economic performance. Further, it is a profound construct that assists in ecological production. In manufacturing, the customer has a desire for recycling goods, cleaner production, and eco-friendly packaging. Customers usually pay a premium price for products that benefit the environment [44]. Inevitably, this phenomenon makes the organization focus on eco-friendly activities that satisfy the customer’s needs, ultimately influencing the firm’s FP [1].

With this, the IGSCM significantly elevating the firm’s FP also compels the business towards achieving competitiveness. In manufacturing, the frequent market changes have made the firms focus on internal business functions. The supply chain management activities encourage the firms to work towards environmental protection. It helps organizations comply with environmental standards, eventually gaining a dominant market position [45]. The IGSCM makes the firms align their ecological goals with their performance. It encourages them to invest in pollutant-free activities [1]. It makes the firms adopt processes, technologies, and resources that are strategically more important to gain a green competitive edge [46]. The company gains competitive benefits when its products reach a novelty that is hard to replicate by the competitors. Hence, in this regard, IGSCM plays a critical role in providing a superior competitive position to the firms [47].

For manufacturing organizations, developing a green supply chain management has always been a challenging task. But in recent years, the growing environmental awareness has urged supply chain organizations improve the EVP [48]. The IGSCM is seen as a synergistic combination of firms' financial and environmental management [49]. In manufacturing, the coordinated activities monitoring compliance with the ecological requirements help the firms to improve EVP [23]. Given the focal point of the stakeholders, the IGSCM meets the green demands of the customers by satisfying their desire for eco-friendly products. The dominant market pressure makes the organizational members pay more attention to its internal affairs, resources, and practices that help them handle environmental issues [9]. The green culture embedded within the organization encourages proactive manufacturers to extend their green efforts outside of the firm proximity in the shape of greener offerings [50]. Indeed, promoting environment-friendly practices can be subject to high EVP from the stakeholder side [51]. Hence, based on the presented argument, we conclude that the IGSCM positively influences FP, GCA, and EVP.

H2(a1) Internal green supply chain management have positive and significant impact on financial performance.

H2(a2) Internal green supply chain management has positive and significant impact on green competitive advantage.

H2(a3) Internal green supply chain management has positive and significant impact on environmental performance.

### 2.3 Green technology adoption

Over the years, climate change has made firms alter their economic structure [2]. Companies that see the new technological advancement as an advantage raise their output [9]. Green technology adoption refers to the utilization of eco-friendly digital tools in business operations [52]. It involves the application of new tools in energy conservation [53] and firms' financial performance. The GTA is a proactive strategy to combat climate change. Greener manufacturing makes the companies use renewable energy resources that biologically nourish the firm’s FP [54].

Green developments pave the solid foundation for the manufacturing industry. In an attempt to integrate green digital technologies to facilitate product design, GTAs have become a need for the global manufacturing industry. Utilizing the modern technological developments in green manufacturing is today a challenging endeavor. Technical progress is the most needed when it comes to satisfying the stakeholders' needs. In the last few years, greener technologies have made the firms ensure stakeholders' requirements, thus achieving green competitiveness [55]. It makes the firms offer non-substitutable products [56]. It makes the enterprise differentiate itself from the rival firms [52]. Therefore, to ensure prolonged rapid economic growth supporting environmental protection, the GTA is a vital construct leading to firms' financial performance [57].

Undoubtedly, enterprises updating their resources achieve GCA by adopting a greener technological approach [58]. Accordingly, Sarfraz et al. [9] study shows that GTA, also affects the firm’s EVP. Today, the manufacturing economy has made the technical progress to deem the depressing effect of ecology. Spanning from solar panels to the advanced environmental management system, the GTA has conserved the natural environment by introducing more sustainable alternatives. It has made the firms enjoy the technical boost that leads to improved environmental performance [59]. GTA encourages firms to go greener in business operations, design, and production towards exhibiting pollution-controlled performance [60]. This cleaner production technology ensures the firms' EVP by reducing excessive energy consumption. Given the increased awareness of environmental performance, the leading technological applications have emerged as the profound tool facilitating the firm’s greener performance. As intelligent manufacturing aims to transform and update the production cycle, the GTA has become a global trend promoting EVP [61]. Hence, in line with these studies, we conclude:

H3(a1) Green technology adoption have positive and significant impact on financial performance.

H3(a2) Green technology adoption have positive and significant impact on green competitive advantage.

H3(a3) Green technology adoption has positive and significant impact on environmental performance.

### 2.4 Green competitive advantage and environmental performance

Today, resource constraints and environmental pollution have placed excessive burdens on manufacturing firms. These undesirable consequences have severely pressurized the firms to embrace new ecological tools in response to the growing vulnerabilities. The green competitive advantage has emerged as a vital tool for increasing the firms' superiority [62]. The competitive advantage enables the companies to generate a product or service that is difficult to imitate by the competitors. Keeping this viewpoint in mind, the GCA helps the company to produce eco-friendly products that are difficult to replicate. The GCA facilitates the firms' activities that encourage energy conservation and waste reduction. It accelerates the firm’s capacity and ability to compete internationally, ultimately influencing firms' EVP [63]. Therefore, supporting the previous literature, we conclude that achieving the GCA makes the company improve EVP at a reduced cost [64]. So, from this, we speculate:

H4 Green competitive advantage has positive and significant impact on environmental performance.

An environmentally friendly business considers the firm’s environmental performance more than FP. This ecological awareness makes them develop capabilities that drive the firms' EVP. In the changing market dynamics, these capabilities are an analytical tool that empowers the manufacturing business to remain competitive. GDC is a winning strategy that cultivates green competitiveness [65]. The GCA is a unique position that the companies enjoy if they offer environmentally friendly goods and services besides their competitors. The environmental concerns for greener performance and competition make the GDC a promising solution to achieving the ecological goals. It is argued that the green dynamic capabilities viewed as an environmental initiative facilitate the firm GCA and EVP [66]. The relationship between the GDC, GCA, and EVP can be highly observed in a manufacturing setup, where these green considerations are seen to support firms' improved EVP and competitiveness [67]. In manufacturing, the GCA signifies an increase in green skills and competence, which enhances the firm’s ecological performance. Indeed, the GDC is a strategic tool that makes the firm achieve GCA, thus contributing to the firm’s EVP [32].

Furthermore, in prior literature, the concept of IGSCM has also been recognized as the most profound tool in enhancing firms' EVP and GCA. The IGSCM increases the firms' desire for superior competitiveness [68]**.** In an SCM, the organization coordinates and adopts the green parameters that increase the firms' decision-making [69], performance, and competitiveness. As in manufacturing, the stakeholder preferences keep changing. Therefore, producing ecological products through adopting innovative practices can make firms maintain a lead. The manufacturing industry facing considerable environmental pressure today makes firms adopt practices that enhance their EVP [70]. The IGSCM revolutionizes business processes, procedures, and models. It progressively converts traditional business practices into greener activities, profoundly becoming an effective strategy for securing GCA and environmental performance [71].

Accordingly, smart digital technologies equip manufacturing firms to accomplish ecological goals by nurturing the firms' EVP. Digital technologies complementing the firm’s greener processes are conducive to improving the EVP. In manufacturing, green technology ensures the production of environmentally friendly goods. Green technology is a science that makes firms gain a superior market position. The companies incorporating the GTA improve EVP and GCA. The GTA helps the companies gain renewable energy resource that lowers the value of the rival firms. It makes the firm achieve a superior differentiating edge [14] through cleaner production. Given the explanation, Shahzad et al. [52] state that companies reduce waste production by adopting new ecological business practices. The GTA’s interaction with the environment improves the firm’s environmental performance [72]. As the protection and restoration of biodiversity requires innovative technologies [60], this integration is a unique one supporting the firm’s performance [73] and competitive advantage. Hence, in line with these arguments, the hypotheses conclude:

H4(a) Green competitive advantage mediates the relationship between Green dynamic capabilities and environmental performance.

H4(b) Internal green supply chain management mediate the relationship between Internal green supply chain management and environmental performance.

H4(c) Green competitive advantage mediates the relationship between green technology adoption and environmental performance.

### 2.5 Green competitive advantage and financial performance

Today, the competition is all about gaining market share. The organization with the highest market share can charge a high price for their product. When an enterprise offers an additional benefit that others don’t, it tends to gain a superior competitive advantage. Going green is a philosophy in supply chain management that enables firms to achieve a green competitive advantage. With green competitive advantage comes many benefits, such as firms' long-term synergistic developments and economic growth, which greatly assist the firm’s performance [7]. As the success of today’s firms depends on the highest returns, sales, and market share, manufacturing organizations are massively embracing new techniques for supporting the firms' FP [63]. This financial aspect encourages the firms to accomplish economic objectives. In green manufacturing, competitiveness is the motivator for the firms FP [74]. The GCA leads to gaining long-term profits. The GCA is what makes the enterprise achieve economic gains. Therefore, today, it has become a need for firms to raise profits. In this regard, the GCA has emerged as a strategic tool for improving the firms' FP. Altogether, based on this, we conclude:

H5 Green competitive advantage has positive and significant impact on financial performance.

In the current environment of fierce competition, enterprises need to integrate the resources that improve the firm’s performance. The core component to improving the firm’s financial performance is to focus on environmental factors. In this regard, the GDC is an effective adjustment that increases the firm’s FP. The GDC brings fruitful economic results for many businesses [28]. It helps the companies gain high returns and sales. The GDC enables firms to secure a position in the market [32], which raises firms' profits. Based on this, Chang et al. [75] state that to achieve competitive advantage and performance, today’s organizations are widely focusing on developing green dynamic capabilities for enhancing the firm’s financial performance. Companies with robust GDCs take advantage of unique positions and performance. The green dynamic capabilities intensify the employees' green skills and behavior, making firms gain GCA [76] and financial victory. Indeed, the GDC is an essential driver of firms' long-term performance and competitiveness. As GCA is hard to replicate, the GDC adds value to the firm’s traditional practices, which improve firms' FP and competitiveness [15].

However, in manufacturing, the pressure for sustainable skills, processes, and production is the key to higher economic output [77]. The supply chain green management is viewed as a profound construct in achieving financial goals and competitiveness. In the current period of turbulence, manufacturing firms are forced to strengthen their internal system for superior FP [70]. For example, it has become vital for firms to reduce their operating cost by colossally lowering the value of the competitor’s businesses [78]. The internal GCMS translates the firms' competitiveness into financial gain [47]. As environmentally friendly practices considerably emphasize using cleaner energies, IGSCM provides a superior competitive advantage to the firm, thus influencing FP [79].

Noticeably, in the world of the technological frontier, the greater the benefit, the more firms gain GCA. Today, concerning the booming green economy, eco-friendly technologies have become a significant way to accomplish green competitiveness and profitability. In manufacturing, the GTA led the firms to become cost-effective [30], efficient, and environmentally responsible [45]. The green technology ultimately reaps the benefit of the ongoing climate change. These existing damages cause the firms to restore the organization’s position and performance. The organizations addressing the ecological issues with the GTA gain a superior ecological edge that is difficult to replicate by the other firms [80]. With this, the GTA has received considerable appreciation in production. Green technology allows firms to earn more revenue by offering green products and services [81]. Green products occupy more market share than any other product [82]. It provides financial incentives to businesses, enabling the firms to save money and resources, thus acquiring GCA. However, understanding this connotation and appreciating the green technological solutions can make manufacturing firms gain maximum profits at a low cost [83]. Hence, in manufacturing, the GTA is noted to be a promising tool for increasing the GCA and FP. Therefore, we conclude:

H5(a) Green competitive advantage mediates the relationship between green dynamic capabilities and financial performance.

H5(b) Green competitive advantage mediates the relationship between internal green supply chain management and financial performance.

H5(c) Green competitive advantage mediates the relationship between green technology adoption and financial performance.

### 2.6 The moderating role of green knowledge acquisition and industry dynamism

The debate on the growing environmental issues has gained much ecologist attention. With this, continuous efforts are taken by the stakeholders to create awareness about the dwindling natural resources and the depleting environment worldwide. Because of the increased environmental awareness, today, firms are experiencing considerable stakeholder pressure for environmental protection. In boosting the confidence of the stakeholders, knowledge management is the key to strengthening the firm’s abilities [84]. Knowledge is the core competence that uplifts the firm’s skills, abilities, and resources that provide GCA [85]. There are many ways through which the company can acquire knowledge. The most valuable knowledge is the one that helps the firms develop the resource that brings numerous economic profits and return volumes. In manufacturing, green knowledge encourages novel ideas, thoughts, and content. The accumulation of green knowledge drives the firms' innovation, which increases the firms' performance. Environmental knowledge fosters the firm’s innovativeness and competitiveness [86]. It transforms traditional business practices into new, valuable, and environmentally friendly processes, which is the requisite for achieving GCA. Environmental knowledge is a strategic resource [87] that allows the company to gain a superior differentiating edge [26]. Following this, several studies contribute that the aspects of the GKA in China eradicate the economic resource constraints [39]. It enhances the firms' competitiveness and performance. Since creating new knowledge is essential for GCA [88], the new knowledge supports developing eco-friendly cultures that elevate the firm’s FP. Hence, based on the previous studies, we conclude:

H6 Green knowledge acquisition moderates the relationship between green competitive advantage and financial performance.

With the rising use of fossil fuel, the Industrial Revolution has led worldwide economies to experience an increase in environmental vulnerabilities, which is the root cause of the firm’s downfall. The ecological issues transcending the national borders have primarily made the manufacturing firms tackle the problem of the changing industry dynamics. Industrial dynamics refers to the unpredictability and volatility of market changes [25]. The manufacturing industry is a fast-growing sector where stakeholders' demands keep changing. Today, these high market fluctuations have made companies shift from traditional business methods to green practices [89], which is essential for gaining GCA. Developing eco-friendly products and minimizing the environmental impact harmonizes the firms' performance with stakeholders' expectations. However, in the last few years, natural resource scarcity has tremendously grown, thus putting the firm’s EVP at risk [2]. This trend has always been the center of the ecologist’s attention as it colossally influences biodiversity and ecological modernization. The industry fluctuations have emphasized managing the environmental burden, thus contributing to GCA and EVP. This environmental convergence requires firms to invest in GCA, which is the pathway to gaining an improved EVP [16]. With the firms demanding constant innovation, it has become imperative to adapt to the rapidly changing market conditions to gain GCA and EVP. Hence, based on these studies, we conclude:

H7 Industry dynamism moderate the relationship between green competitive advantage and environmental performance.

**Fig. 1:**
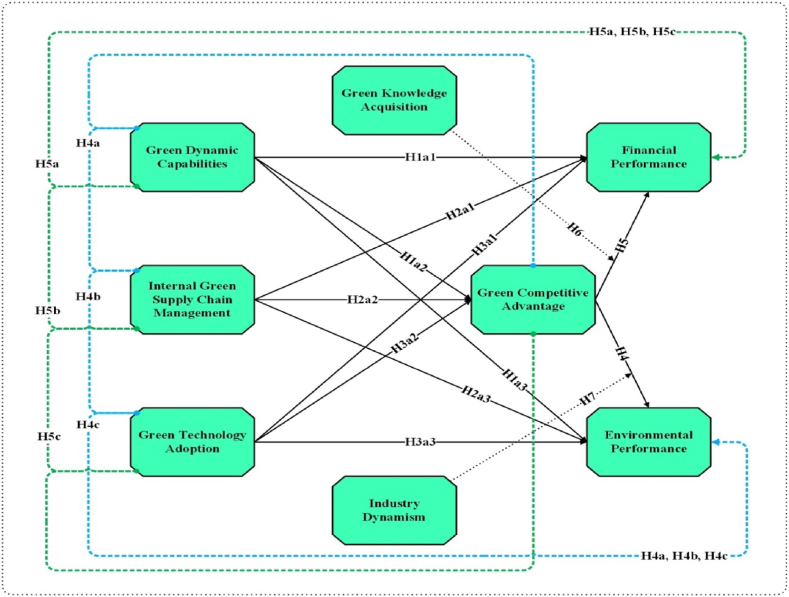
Conceptual framework.

Fig. 1 shows study variable hypothesis (Independent variables, dependent variables, Mediator and Moderator variables).

## 3 Methodology

The study’s target group consisted of employees from the Chinese manufacturing companies. The participant selection was based on accessibility, given the ease in reaching out to this group. During data collection, the employees chosen are those most readily available, straightforward to engage, and willing to participate. In the initial stages of this study, sample collection posed a challenge due to unique circumstances within the manufacturing industry. Given the economic instability, data gathering was executed through online surveys administered via a web-based interface. This method allowed respondents to provide their input independently, reducing any direct interaction between researchers and participants. Before engaging in the survey, all participants received assurances of the strict confidentiality and anonymity of their responses. For the data analysis, we employed the Structural Equation Modeling (SEM) method. A sample size ranging from 200 to 250 valid entries is deemed suitable. Data gathering transpired during March and July 2023.

Table 1 shows the demographic details of 471 participants. Of these, 61.8% are male and 38.2% female. Age-wise, the largest group is 18–30 years at 39.9%, followed by 31–40 years at 29.3%. In terms of education, master’s degree holders form the majority at 43.7%, closely followed by Bachelors at 39.3%. Position-wise, most are non-administrative employees at 60.7%. By firm type, there’s a near-equal split between textile and chemical products employees, at 32.3% and 33.3% respectively, with fertilizers at 16.1%.

**Table 1:** Demographic characteristics.

Items	Frequency (N = 471)	(%)
**Gender**		
Male	291	61.8
Female	180	38.2
**Age**		
18–30 Years	188	39.9
31–40 Years	138	29.3
41–50 Years	73	15.5
51–60 Years	57	12.1
Over 60 Years	15	3.2
**Education**		
Bachelors	185	39.3
Masters	206	43.7
M.Phil./Others	80	17
**Position**		
Head of Department/Manager	29	6.2
Administrative Employee	156	33.1
Non-Administrative Employee	286	60.7
**Firm Type**		
Textile	152	32.3
Chemical Products	157	33.3
Fertilizers	76	16.1

### 3.1 Common method bias

This research also applied the common method bias using Harman’s single-factor approach. The variance extracted using one factor is 12.779%, less than 50%, indicating no common method bias in this study Podsakoff et al. [90].

## 4 Results

Table 2 provides an analysis of reliability and validity for various constructs. Each construct’s items, their respective loadings, Cronbach’s alpha, composite reliability (CR), and average variance extracted (AVE) values are presented. For example, the construct GDC, the items GDC_1 to GDC_5 have loadings ranging from 0.780 to 0.824, with an alpha of 0.864, CR of 0.902, and AVE of 0.648. The IGSCM construct has items IGSCM_1 to IGSCM_7 with loadings between 0.755 and 0.798, an alpha of 0.888, CR of 0.912, and an AVE of 0.597. The GTA construct, encompassing items GTA_1 to GTA_5, shows loadings from 0.780 to 0.802, an alpha value of 0.851, CR of 0.894, and an AVE of 0.627. For GCA, with items from GCA_1 to GCA_4, the loadings range between 0.800 and 0.825, accompanied by an alpha of 0.831, CR of 0.888, and an AVE of 0.664. GKA’s items, GKA_1 to GKA_6, have loadings varying from 0.616 to 0.871, with an alpha of 0.849, CR of 0.874, and an AVE of 0.539.

**Table 2:** Reliability and validity analysis.

Construct	Items	Loading	Alpha	CR	AVE
			>0.7	>0.7	>0.5
GDC	GDC_1	0.824	0.864	0.902	0.648
	GDC_2	0.780			
	GDC_3	0.816			
	GDC_4	0.806			
	GDC_5	0.798			
IGSCM	IGSCM_1	0.762	0.888	0.912	0.597
	IGSCM_2	0.755			
	IGSCM_3	0.784			
	IGSCM_4	0.798			
	IGSCM_5	0.762			
	IGSCM_6	0.775			
	IGSCM_7	0.773			
GTA	GTA_1	0.793	0.851	0.894	0.627
	GTA_2	0.802			
	GTA_3	0.791			
	GTA_4	0.792			
	GTA_5	0.780			
GCA	GCA_1	0.800	0.831	0.888	0.664
	GCA_2	0.825			
	GCA_3	0.817			
	GCA_4	0.816			
GKA	GKA_1	0.732	0.849	0.874	0.539
	GKA_2	0.616			
	GKA_3	0.871			
	GKA_4	0.740			
	GKA_5	0.683			
	GKA_6	0.738			
ID	ID_1	0.704	0.787	0.858	0.603
	ID_2	0.756			
	ID_3	0.850			
	ID_4	0.788			
FP	FP_1	0.811	0.862	0.900	0.644
	FP_2	0.813			
	FP_3	0.797			
	FP_4	0.801			
	FP_5	0.790			
EVP	EVP_1	0.805	0.867	0.904	0.653
	EVP_2	0.812			
	EVP_3	0.801			
	EVP_4	0.834			
	EVP_5	0.787			

Table 3 provides insights into the discriminant validity of various constructs, utilizing both the Fornell-Larcker criterion and HTMT. The correlation between EVP and FP is 0.556, between EVP and GCA is 0.537, and so on. It’s important to note that for adequate discriminant validity using the Fornell-Larcker criterion, the diagonal values (square root of AVE) for a construct should ideally be greater than its off-diagonal values (correlations with other constructs). This pattern continues for all the constructs in the table, giving a comprehensive view of how each construct relates to others and its own discriminant validity.

**Table 3:** Discriminant validity (Fornel Larcker & HTMT).

Constructs	1	2	3	4	5	6	7	8
1. EVP	**0.808**	0.642	0.633	0.683	0.153	0.672	0.170	0.688
2. FP	0.556	**0.802**	0.575	0.695	0.080	0.662	0.115	0.662
3. GCA	0.537	0.487	**0.815**	0.612	0.188	0.614	0.205	0.658
4. GDC	0.592	0.600	0.520	**0.805**	0.149	0.686	0.140	0.688
5. GKA	−0.148	−0.087	0.133	−0.148	**0.734**	0.134	0.309	0.115
6. GTA	0.579	0.568	0.518	0.590	−0.135	**0.792**	0.121	0.679
7. ID	−0.150	−0.105	0.156	−0.120	0.247	−0.104	**0.776**	0.160
8. IGSCM	0.605	0.579	0.567	0.604	−0.113	0.592	−0.139	**0.773**

**Note:** “Values on the diagonal (italicized) represent the square root of the average variance extracted, while the off diagonals are correlations”.

**Fig. 2:**
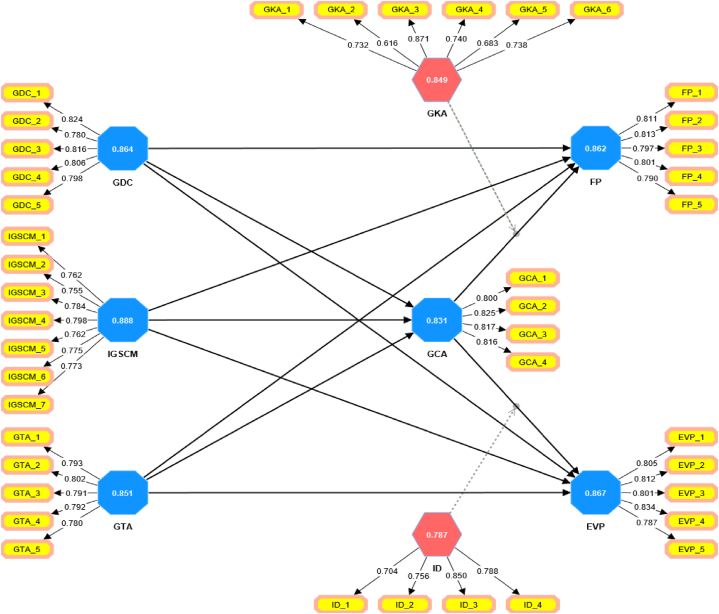
Assessment of measurement model.

Table 4 presents the Variance Inflation Factor (VIF) analysis for various constructs. The VIF values provide insights into the level of multicollinearity between predictor variables. It’s evident from the presented values that there are no instances of high multicollinearity among the constructs since all VIF values are well below the threshold of 3.3. Fig. 2 is graphical representation of assessment of measurement model.

**Table 4:** Variance inflation factor (VIF) analysis.

Constructs	1	2	3	4	5	6	7	8	9	10
1. ECINO										
2. EP										
3. GBS	2.026	2.045								
4. GHC	1.918	1.949	1.811							
5. GRC		1.144								
6. GSC	1.887	1.891	1.771							
7. SFP	1.196									
8. TA	2.115	2.081	1.818							
9. GKA x GCA		1.249								
10. ID x GCA	1.284

Table 5 elucidates the results of hypotheses testing for direct effects. The presented relationships, standard beta coefficients, standard errors, t-values, and significance levels are all indicative of the strength and reliability of the proposed hypotheses. For the hypothesis H1a1, which posits a direct relationship between GDC and FP, the standard beta coefficient is 0.266 with a standard error of 0.044, leading to a t-value of 6.055.

**Table 5:** Hypotheses testing direct effect.

Hypothesis	Direct Relationships	Std. *Beta*	Std. Error	T- Values	P- Values
H1(a1)	GDC → FP	0.266	0.044	6.055	***
H1(a2)	GDC → GCA	0.203	0.055	3.686	***
H1(a3)	GDC → EVP	0.204	0.043	4.781	***
H2(a1)	IGSCM → FP	0.190	0.044	4.301	***
H2(a2)	IGSCM → GCA	0.321	0.050	6.424	***
H2(a3)	IGSCM → EVP	0.193	0.048	4.052	***
H3(a1)	GTA → FP	0.198	0.045	4.358	***
H3(a2)	GTA → GCA	0.208	0.048	4.320	***
H3(a3)	GTA → EVP	0.179	0.042	4.272	***
H4	GCA → EVP	0.258	0.045	5.705	***
H5	GCA → FP	0.155	0.055	2.832	**

*Indicates significant paths: **p < 0.01, ***p < 0.001.

H1a2 examines the relationship between GDC and GCA, the beta coefficient is 0.203, the standard error is 0.055, and the t-value is 3.686. This too is statistically significant at the p < 0.001 level. This pattern of significant relationships at the p < 0.001 level continues for the hypotheses H1a3 through H3a3, as all these relationships have t-values which affirm their significance. Hypothesis H4, which postulates a direct relationship between GCA and EVP, has a standard beta coefficient of 0.258, a standard error of 0.045, and a significant t-value of 5.705.

Lastly, hypothesis H5 suggests a relationship between GCA and FP with a beta coefficient of 0.155, a standard error of 0.055, and a t-value of 2.832. This relationship is significant at the p < 0.01 level, as indicated by the ‘**’. In summary, all the hypotheses presented in Table 5 demonstrate statistically significant relationships.

**Fig. 3:**
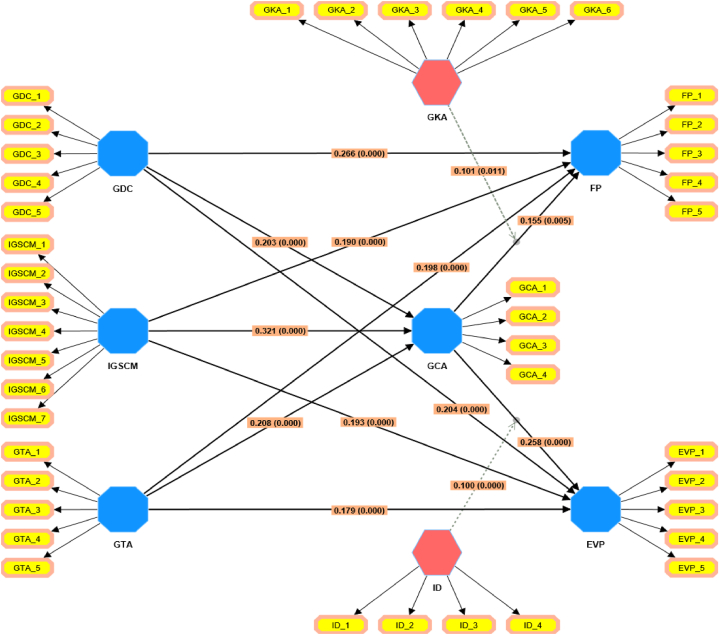
Graphical representation of the structural model.

Table 6 presents the results of hypotheses testing for mediation effects. For hypothesis H4a, which asserts a mediation effect of GCA between GDC and FP, the standard beta coefficient is 0.032 with a standard error of 0.015. This leads to a t-value of 2.124, which is statistically significant at the p < 0.05 level, as denoted by the ‘*’. Hypothesis H4b, suggesting a mediating effect of GCA between IGSCM and FP, shows a beta coefficient of 0.050, standard error of 0.019, and a t-value of 2.558. This result is significant at the p < 0.01 level, indicated by the ‘**’. For H4c, positing a mediation effect of GCA between GTA and FP, the beta value is 0.032, with a standard error of 0.014, yielding a t-value of 2.233. This is significant at the p < 0.01 level. Turning to hypothesis H5a, which suggests GCA mediates the relationship between GDC and EVP, it has a beta coefficient of 0.053, standard error of 0.018, and a t-value of 2.873. This relationship is statistically significant at the p < 0.05 level. Hypothesis H5b, which proposes a mediation effect of GCA between IGSCM and EVP, displays a beta of 0.083, standard error of 0.019, and a t-value of 4.439. This result is significant at the p < 0.05 level. Finally, H5c, postulating GCA as a mediator between GTA and EVP, shows a beta value of 0.054, standard error of 0.017, and a significant t-value of 3.247 at the p < 0.001 level, as represented by ‘***’. In summary, all mediated relationships presented in Table 6 are statistically significant, with varying levels of significance across p < 0.05, p < 0.01, and p < 0.001. Fig. 3 is graphical representation of the structural model.

**Table 6:** Hypotheses testing Mediation Effect.

Hypothesis	Direct Relationships	Std. *Beta*	Std. Error	T- Values	P- Values
H4(a)	GDC → GCA → FP	0.032	0.015	2.124	*
H4(b)	IGSCM → GCA → FP	0.050	0.019	2.558	**
H4(c)	GTA → GCA → FP	0.032	0.014	2.233	**
H5(a)	GDC → GCA → EVP	0.053	0.018	2.873	*
H5(b)	IGSCM → GCA → EVP	0.083	0.019	4.439	*
H5(c)	GTA → GCA → EVP	0.054	0.017	3.247	***

*Indicates significant paths: *p < 0.05, **p < 0.01, ***p < 0.001.

**Fig. 4:**
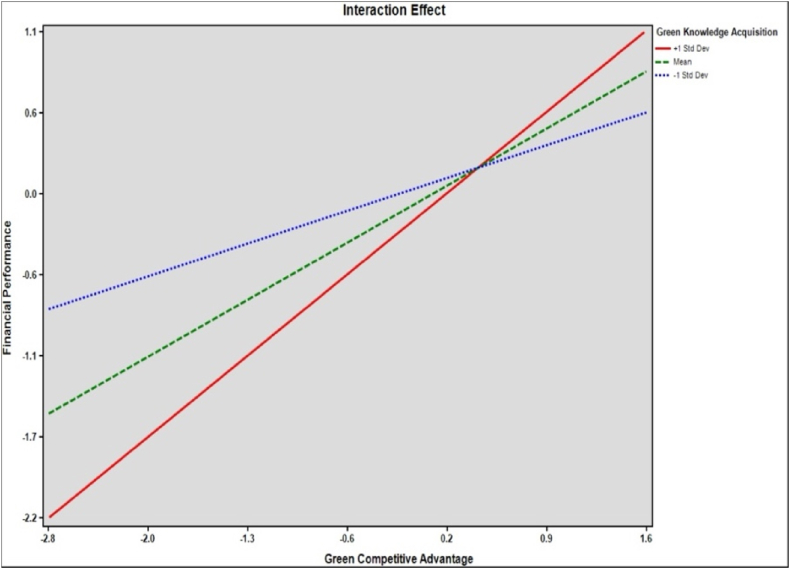
Interaction GKA x GCA → FP.

**Fig. 5:**
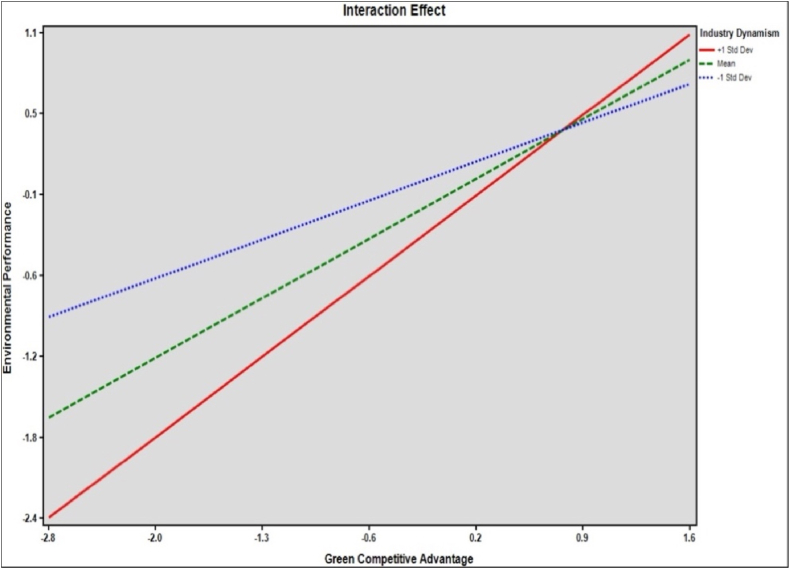
Interaction ID x GCA → EP.

Table 7 presents the results of hypotheses testing for moderation effects. The table outlines the relationships, standard beta coefficients, standard errors, t-values, p-values, and the effects of the moderator at different levels. For hypothesis H6, which proposes an interaction effect of GKA and GCA on FP, the beta coefficient is 0.101, with a standard error of 0.039, leading to a t-value of 2.556. The p-value for this interaction is 0.011. Hypothesis H7 suggests an interaction effect of ID and GCA on EVP. The beta coefficient for this interaction is 0.100, with a standard error of 0.023, resulting in a t-value of 4.341. The p-value is 0.000, indicating a highly significant relationship. The table further breaks down the effect of the moderator at three different levels: +1 standard deviation (Std Dev), mean, and −1 Std Dev. Fig. 4, Fig. 5 shows moderating results.

**Table 7:** Moderating hypotheses results.

Hypothesis	Interaction Effects	Std. *Beta*	Std. Error	T- Values	P- Values
H6	GKA x GCA - > FP	0.101	0.039	2.556	0.011
H7	ID x GCA - > EVP	0.100	0.023	4.341	0.000
**Level of the Moderator**		**Effects**	**Boot SE**	**LLCI**	**ULCI**
H6	+1 Std Dev	0.771	0.054	0.666	0.876
	Mean	0.541	0.038	0.465	0.617
	−1 Std Dev	0.311	0.047	0.218	0.404
H7	+1 Std Dev	0.805	0.050	0.706	0.904
	Mean	0.597	0.036	0.525	0.668
	−1 Std Dev	0.389	0.046	0.298	0.479

## 5 Discussion

In recent years, novel ecological adoptions have made manufacturing businesses reframe their business activities, potentially improving firms' performance [61]. Thus, concerning this inevitable shift in business, GDCs have profoundly emerged as a strategic tool assisting environmental changes. During our analysis, we found a positive role of GDC in the manufacturing sector. Consistent with the prior literature, our findings showed that GDC has profoundly influenced firms' FP, GCA, and EVP (i.e., H1a (1,2,3)). Iqbal et al. [28] showed that GDC enables firms to tap the opportunities that drives the firms' economic and environmental performance [15]. Furthermore, the prior study shows that GDC also enhances GCA [32]. As the ecological capabilities are difficult to imitate by others, the GDC offers a strong value proposition to the firms that help the companies to go green, thus improving the firm’s performance.

With green capabilities yielding the firms' performance, the IGSCM also adds value to firms' economic structure [91]. As a pillar of the organization, it solves the organization’s environmental problems. It transforms the firm’s supply chain model. Environmental performance is positively correlated with activities, policies, and ecological cross-functional audits [92]. In explaining this notion, Sun et al. [93] state that firms' ecological functions increase the firm’s EVP and competitiveness [47]. Therefore, investigating the IGSCM, our findings confirm the positive role of ICSCM in FP, GCA, and EVP (i.e., H2a (1,2,3)). With this, we also found manufacturing businesses to adopt novel technologies, substantially ensuring their FP and EVP [9] and competitiveness (i.e., H3a (1,2,3)). In green supply chain management, it is essential to eliminate the level of industrial pollution in the ecosystem. The GTA helps manufacturing firms increase their operational efficiencies, substantially improving firms' EVP [94]. The GTA fosters the firms' innovative processes, which certify their FP [95]. The GTA, while mitigating climate change, also increase firms' competitive position [96]. Green technology innovations help the firm maximize economic benefits and competitiveness [97]. This phenomenon is explained in Hassan et al. [56] study, which states that GTA ensures the firm’s high competitiveness. Surprisingly, our study results also show information technology to be a pivotal tool of today’s businesses performance and competitiveness [53].

However, holding the potential of these advanced tools beyond merely mitigating the environmental harm to flourishing the firms' green competitiveness [98], Green competitiveness has helped the company achieve not just ecological goals but also its financial objectives. Through GDC, the firms can achieve GCA and FP [7]. Financial performance is a complex construct that ensures the firm’s performance [77]. The GDC helps the firm achieve a high level of FP [31]. Kalyar et al. [99] suggest that IGSCM enables firms to align their ecological goals with FP. The IGSCM maximizes the firm’s returns and GCA [79]. Given this, our study also found that for reducing the overall effect of ecological degradation on the environment, GTA has emerged as the most profound digital tool, improving the firms' FP. Weighing the role of GTA in manufacturing, Zhang et al. [100] reveal that IT equipment has made the firms deploy energy-efficient products, compelling firms to improve their environmental and financial performance. Hence, based on our findings, we confirm a positive mediation of the GCA (i.e., H4 and H5 (a, b, c)).

Accordingly, with new technologies nurturing the organization’s competitiveness and performance, factors such as global arrangements and market conditions have profoundly affected firms' manufacturing. In recent years, the high competition has made firms focus on cleaner production, thus improving the firm EVP [101]. The fluctuations in the business environment influence the business EVP. Since manufacturing businesses are economic units, governments must take account of the market dynamics to create a positive impact on the natural environment [4]. Green knowledge makes firms respond to the market change. GKA enhances the firms' technical aspects that create value for the businesses. Ecological knowledge plays a crucial role in bringing the firm’s green advantage. Given this, we found that the organization’s learning is the power to achieve GCA [102] and FP. GKA makes the firms respond to the ecological need, which improves the firm’s FP [103]. Altogether, in a rapidly changing environment, green knowledge organization has emerged as the most vital tangible resource that shapes and strengthens the firm’s profitability and competitiveness. Thus, our study confirms a moderating role of GKA and ID as per H6 and H7. To sum up, our study findings show all our evaluations to be relevant, concise, and objective, thus illustrating positive manufacturing results.

## 6 Conclusion

Over the years, mounting environmental depletion has caused detrimental effects on the firm’s performance. China, gaining remarkable achievements, is still not able to combat stringent ecological problems. Hence, in this regard, this study aims to bridge the gap by explaining to the real world the complexities of the Chinese manufacturing sector. In the unsettled manufacturing market, businesses have faced severe competition that has encouraged many firms to focus on their performance. Firms committed to environmental integrity uplift their performance through GDC, IGSCM, and GTA. Given this, this study investigated the effect of GSC, IGSCM, and GTA on the firms' FP and EVP under the mediated moderated model (i.e., GCA and ID). The manufacturing firms taking proactive measures engage in green activities to gain GCA. Green manufacturing makes companies adopt renewable energies that encourage the production of recycled goods and technology adoption. Our findings showed that GDC, IGSCM, and GTA positively influence the firms' FP and EVP. It also confirmed the significant mediated and moderated role of GCA, GKA, and ID nexus to GCA and performance (i.e., FP and EVP). All the results were accepted and supported, thus suggesting directions for policymakers, manufacturers, practitioners, and business firms. Altogether, this study’s findings are significant ones that make the firms invest in programs that are difficult to imitate by the competitors. This encouragement leads industrial companies to pay attention to the rising environmental issues influencing the firms' competitiveness and performance. Indeed, we hope that the framework adopted in this study positively influences the firms' performance and competitive advantage. We wish that this study works as a guide for future scholars, thus bringing fruitful results in the manufacturing world.

### 6.1 Limitation and future recommendations

Significantly, the current study presents implications for stakeholders, practitioners, and policymakers. With that, a few study challenges need to be overcome to expand the study outcomes. The study can be extended in several ways. Firstly, clear clarification is required on the relationship between digitalization and green dynamic capabilities in modern business. Traditional businesses may face several challenges in implementing green technology supporting the firms' capabilities. Therefore, it is vital for future studies to deeply explore the current relationships from diverse viewpoints. This act will provide a more nuanced understanding of the relationship required to achieve the EVP, FP, and GCA. The prior literature showed that it is essential to reduce the complexity of digitalization to gain GCA. Therefore, future studies can explore digitalization, green dynamic capability, and technology from diverse perspectives. Understanding this relationship will enable the organization to benefit from the new information. It will help them exploit the new opportunities by adopting GDC, IGSCM, and GTA, ultimately influencing EVP, FP, and green competitiveness.

With this, it is suggested that future research must examine the internal and external green supply chain management practices to gain better EVP and FP. Our study only investigated the role of the IGSCM. Therefore, it is advised to explore external green supply chain management to widen the scope of the study. In the future, this act will help the managers to focus on their strategic planning process. The manufacturing organizations should have a clear view of environmental preservation. This integration will help them go green in designing their supply chain policies concerning the stakeholders (i.e., employees and suppliers), which will help them achieve a sustainable competitive edge in the business market.

Thirdly, in the current study, limited variables observe the impact on the firm’s performance. Future scholars can include more variables, such as Green Satisfaction, GHRM, and Green Innovation, as moderators and mediators to see the variation in the study results. This initiative will help the managers pay attention to the other factors that can positively drive the firm’s performance. Moreover, this model is deployed in the developed market of China. So, future samples can be taken from different geographical countries (e.g., Pakistan, Romania) to explore the cross-country heterogeneity. Also, it only focuses on a single industry. More industries can be studied to better understand the impact of digitalization and green dynamic capabilities on the business’s sustainable performance and competitiveness. Furthermore, our analysis is restricted to the broad role of digital technology. Therefore, to gain deeper insight, future studies can investigate the role of various technologies, such as Artificial Intelligence, Big Data, IoT, etc., against the firm’s performance (i.e., EVP and FP) and competitiveness.

Thus, by exploring these missing links, organizations can realize the value of the most effective performance improvement. All these recommendations can help the companies to drive their supply chain operations to achieve superior performance results. Indeed, by conducting future research by adopting these perspectives, we can uncover insight information on the complex relationships that have previously not been explored. In an uncertain business environment, it is imperative to provide management practitioners with a better understanding of the digitalization and green dynamic capabilities and resources that demand investment, time, and effort. Hence, this new research will open avenues for managers, organizations, practitioners, and policymakers to consider the influence of these factors on the firms' performance and green competitiveness.

## Data availability statement

The data that support the findings of this study are available from the corresponding author upon reasonable request.

## Funding statement

The study was funded by Doctoral Initiation Project of 10.13039/501100010844Hanshan Normal University (QD202109) and Behavioral Science Research Center of 10.13039/501100010844Hanshan Normal University (PSB2101).

## Ethical statement

All participants gave their informed consent for inclusion before they participated in the study. All procedures performed were by the ethical standards as laid down in the 1964 Declaration of Helsinki and its later amendments or comparable ethical standard.

## CRediT authorship contribution statement

**Zeying Li:** Writing – review & editing, Software, Resources, Funding acquisition, Data curation. **Saad Rasool:** Writing – original draft, Formal analysis, Conceptualization. **Mustafa Fedai Cavus:** Writing – review & editing, Validation. **Waseem Shahid:** Writing – review & editing, Software, Funding acquisition.

## Declaration of competing interest

The authors declare that they have no known competing financial interests or personal relationships that could have appeared to influence the work reported in this paper.

## Figures and Tables

**Table:** 

Construct Name	Green Dynamic Capabilities
GDC_1	The firm is able to exploit, integrate, combine, create, acquire, share, and convert new environmental technology
GDC_2	The firm is able to effectively deploy resources for the development of green innovations
GDC_3	The firm is able to effectively coordinate employees to generate green knowledge
GDC_4	The firm is able to effectively manage and assimilate specialized environmental technology within the firm
GDC_5	The firm can quickly observe the environment and recognize new environmental opportunities
	**Internal Green Supply Chain Management**
IGSCM_1	We have increased the usage of environmentally friendly raw materials and components
IGSCM_2	We have designed our products and/or services so that their materials can be recycled
IGSCM_3	Being environmentally conscious is an integral part of our corporate culture.
IGSCM_4	We plan the deliveries of the company to minimize the environmental impacts.
IGSCM_5	We utilize green marketing for our products and/or services.
IGSCM_6	We conduct internal environmental audits to ensure that products and/or services meet the environmental goals.
IGSCM_7	We do cross-functional cooperation for mitigating environmental impacts.
	**Green Technology Adoption**
GTA_1	Eco-technology changes rapidly in our firm
GTA_2	We are eco-technologically competitive
GTA_3	We use up-to-date/new technology in the process
GTA_4	We are fast in adopting the latest technological innovations
GTA_5	We use cleaner technologies
	**Green Competitive Advantage**
GCA_1	Compared to our major competitors, we have the competitive advantage of low-cost environmental management or green innovation
GCA_2	e quality of the green products or services we offer is better than that of our major competitor
GCA_3	We are more capable of environmental R&D and green innovation than our major competitors
GCA_4	We are more capable of environmental management than our major competitors
	**Financial Performance**
FP_1	Our market share grew faster than our competitors in the three years after we adopted a digital servitization strategy
FP_2	Our profit grew faster than our competitors in the three years after we adopted a digital servitization strategy
FP_3	Our return on total assets grew faster than our competitors in the three years after we adopted a digital servitization strategy
FP_4	Our overall competitive position grew faster than our competitors in the three years after we adopted a digital servitization strategy
FP_5	Our number of successful new product/service introductions grew faster than our competitors in the three years after we adopted a digital servitization strategy
	**Environmental Performance**
EP_1	Our organization improved compliance with environmental standards
EP _2	Our organization reduces air emissions
EP _3	Our organization reduces energy consumption
EP_4	Our organization reduces material usage
EP_5	Our organization reduces consumption of hazardous materials
	**Industry Dynamism**
ID_1	Our product and services become outdated
ID_2	Our organization continuously introduces new products and services
ID_3	Our organization introduces new operating processes
ID_4	The customers taste and preferences in our industry changes fast
	**Green Knowledge Acquisition**
GKA_1	Our company acquires know-how related to environmental protection
GKA_2	Our company acquires business knowledge related to environmental protection
GKA_3	Our company acquires knowledge about the environmental effects of products, materials and services
GKA_4	Our company acquires knowledge about environmental regulation
GKA_5	Our company acquires knowledge about environmental management manuals and techniques
GKA_6	Our company acquires knowledge about environmental efficiency

## AbbreviationNomenclature

FP: Financial Performance
EVP: Environmental Performance
IGSCM: Internal Green Supply Chain Management
GCA: Green Competitive Advantage
GKA: Green Knowledge Acquisition
ID: Industry Dynamism
GDC: Green Dynamic Capabilities
GTA: Green Technology Adoption
SEM: Structural Equation Modelling
CR: Composite Reliability
AVE: Average Variance Extracted
Std Dev: Standard Deviation
