# Supplementary material for: Sustaining the future: How green capabilities and digitalization drive sustainability in modern business
Source: Heliyon. 2024 Jan 7;10(1):e24158. doi: 10.1016/j.heliyon.2024.e24158 (PMC10792582; doi:10.1016/j.heliyon.2024.e24158)
Supplement: Multimedia component 1 [file mmc1.docx]

**Questionnaire**

This survey is being conducted to investigate “Sustaining the Future: How Green Capabilities and Digitalization Drive Sustainability in Modern Business”. Please spare your 10-15 minutes to response on following questions. The information that you provide will be kept strictly confidential and will be used for the research only.

**BASIC INFORMATION**

**Name: ___________________________________**

1. **Gender:** (1) Male (2) Female
2. **Age:** (1) 18-30 (2) 31-40 (3) 41-50 (4) 51-60 (5) >60
3. **Education:** (1) Bachelor (2) Master (3) M.Phil./Others
4. **Position** (1) Head of Department/Manager (2) Administrative Employee (3) Non-Administrative Employee
5. **Firm Type**
   - - 1. Automobiles
       2. Textile
       3. Chemical Products
       4. Fertilizers
6. **Indicate (✓) your level of agreement with the following statements.**

**1=SD:** Strongly Disagree, **2**=D**A:** disagree, **3 = N:** Neutral, **4= SA:** Agree, **5= SA:** Strongly Agree

| Sr.No | **Statement** | **Strongly disagree** | **Disagree** | **Neutral** | **Agree** | **Strongly agree** |
| --- | --- | --- | --- | --- | --- | --- |
| Construct Name | **Green Dynamic Capabilities** | | | | | |
| Paper | **Understanding Corporate Green Competitive Advantage through Green Technology Adoption and Green Dynamic Capabilities: Does Green Product Innovation Matter?** | | | | | |
| GDC_1 | The firm is able to exploit, integrate, combine, create, acquire, share, and convert new environmental technology | SD | DA | N | A | SA |
| GDC_2 | The firm is able to effectively deploy resources for the development of green innovations | SD | DA | N | A | SA |
| GDC_3 | The firm is able to effectively coordinate employees to generate green knowledge | SD | DA | N | A | SA |
| GDC_4 | The firm is able to effectively manage and assimilate specialized environmental technology within the firm | SD | DA | N | A | SA |
| GDC_5 | The firm can quickly observe the environment and recognize new environmental opportunities | SD | DA | N | A | SA |
| Construct Name | **Internal GSCM** | | | | | |
| Paper | **Firm performance and customer-driven green supply chain management** | | | | | |
| IGSCM_1 | We have increased the usage of environmentally friendly raw materials and components | SD | DA | N | A | SA |
| IGSCM_2 | We have designed our products and/or services so that their materials can be recycled | SD | DA | N | A | SA |
| IGSCM_3 | Being environmentally conscious is an integral part of our corporate culture. | SD | DA | N | A | SA |
| IGSCM_4 | We plan the deliveries of the company to minimize the environmental impacts. | SD | DA | N | A | SA |
| IGSCM_5 | We utilise green marketing for our products and/or services. | SD | DA | N | A | SA |
| IGSCM_6 | We conduct internal environmental audits to ensure that products and/or services meet the environmental goals. | SD | DA | N | A | SA |
| IGSCM_7 | We do cross-functional cooperation for mitigating environmental impacts. | SD | DA | N | A | SA |
| Construct Name | **Green Technology Adoption** | SD | DA | N | A | SA |
| Paper | **Understanding Corporate Green Competitive Advantage through Green Technology Adoption and Green Dynamic Capabilities: Does Green Product Innovation Matter?** | | | | | |
| GTA_1 | Eco-technology changes rapidly in our firm | SD | DA | N | A | SA |
| GTA_2 | We are eco-technologically competitive | SD | DA | N | A | SA |
| GTA_3 | We use up-to-date/new technology in the process | SD | DA | N | A | SA |
| GTA_4 | We are fast in adopting the latest technological innovations | SD | DA | N | A | SA |
| GTA_5 | We use cleaner technologies | SD | DA | N | A | SA |
| Construct Name | **Green Competitive Advantage** | | | | | |
| Paper | **Understanding Corporate Green Competitive Advantage through Green Technology Adoption and Green Dynamic Capabilities: Does Green Product Innovation Matter?** | | | | | |
| GCA_1 | Compared to our major competitors, we have the competitive advantage of low-cost environmental management or green innovation | SD | DA | N | A | SA |
| GCA_2 | e quality of the green products or services we offer is better than that of our major competitor | SD | DA | N | A | SA |
| GCA_3 | We are more capable of environmental R&D and green innovation than our major competitors | SD | DA | N | A | SA |
| GCA_4 | We are more capable of environmental management than our major competitors | SD | DA | N | A | SA |
| Construct Name | **Financial Performance** | | | | | |
| Paper | **Unpacking the relationship between digital capabilities, services capabilities, and firm financial performance: A moderated mediation model** | | | | | |
| FP_1 | Our market share grew faster than our competitors in the three years after we adopted a digital servitization strategy | SD | DA | N | A | SA |
| FP_2 | Our profit grew faster than our competitors in the three years after we adopted a digital servitization strategy | SD | DA | N | A | SA |
| FP_3 | Our return on total assets grew faster than our competitors in the three years after we adopted a digital servitization strategy | SD | DA | N | A | SA |
| FP_4 | Our overall competitive position grew faster than our competitors in the three years after we adopted a digital servitization strategy | SD | DA | N | A | SA |
| FP_5 | Our number of successful new product/service introductions grew faster than our competitors in the three years after we adopted a digital servitization strategy | SD | DA | N | A | SA |
| Construct Name | **Environmental Performance** | | | | | |
| Paper | **Environmental MCS package and green intellectual capital influence environmental performance: a mediated‑moderated perspective** | | | | | |
| EP_1 | Our organization improved compliance with environmental standards | SD | DA | N | A | SA |
| EP _2 | Our organization reduces air emissions | SD | DA | N | A | SA |
| EP _3 | Our organization reduces energy consumption | SD | DA | N | A | SA |
| EP_4 | Our organization reduces material usage | SD | DA | N | A | SA |
| EP_5 | Our organization reduces consumption of hazardous materials | SD | DA | N | A | SA |
| Construct Name | **Industry Dynamism** | | | | | |
| Paper | **Big data analytics capability in supply chain agility: The moderating effect of organizational flexibility** | | | | | |
| ID_1 | Our product and services become outdated | SD | DA | N | A | SA |
| ID_2 | Our organization continuously introduces new products and services | SD | DA | N | A | SA |
| ID_3 | Our organization introduces new operating processes | SD | DA | N | A | SA |
| ID_4 | The customers taste and preferences in our industry changes fast | SD | DA | N | A | SA |
| Construct Name | **Green Knowledge Acquisition** | | | | | |
| Paper | **The impact of international experience on firm economic performance. The double mediating effect of green knowledge acquisition & eco-innovation** | | | | | |
| GKA_1 | Our company acquires know-how related to environmental protection | SD | DA | N | A | SA |
| GKA_2 | Our company acquires business knowledge related to environmental protection | SD | DA | N | A | SA |
| GKA_3 | Our company acquires knowledge about the environmental effects of products, materials and services | SD | DA | N | A | SA |
| GKA_4 | Our company acquires knowledge about environmental regulation | SD | DA | N | A | SA |
| GKA_5 | Our company acquires knowledge about environmental management manuals and techniques | SD | DA | N | A | SA |
| GKA_6 | Our company acquires knowledge about environmental efficiency | SD | DA | N | A | SA |
